# Two Cases of Single-Incision Laparoscopic Surgery for Sigmoid Colon and Rectal Cancer in Situs Inversus Totalis

**DOI:** 10.70352/scrj.cr.24-0016

**Published:** 2025-03-25

**Authors:** Mamoru Miyasaka, Koichi Teramura, Shuji Kitashiro, Yuki Okawa, Sho Sekiya, Daisuke Saikawa, Satoshi Hayashi, Yoshinori Suzuki, Masaya Kawada, Yo Kawarada, Kichizo Kaga, Shunichi Okushiba, Satoshi Hirano

**Affiliations:** 1 Department of Surgery, Tonan Hospital, Sapporo, Hokkaido, Japan; 2 Department of Gastroenterological Surgery II, Hokkaido University Faculty of Medicine, Sapporo, Hokkaido, Japan

**Keywords:** situs inversus totalis, single-incision laparoscopic surgery, transanal total mesorectal excision

## Abstract

**INTRODUCTION:**

Situs inversus totalis (SIT) is a rare congenital disorder characterized by the complete inverted transposition of the thoracic and abdominal viscera. This anatomical variation complicates laparoscopic surgery, and there are currently no reports of single-incision laparoscopic surgery (SILS) for patients with sigmoid colon cancer or rectal cancer with SIT.

**CASE PRESENTATION:**

We performed SILS on 2 patients with sigmoid colon and rectal cancers who also had SIT. The first case involved a 64-year-old woman with sigmoid colon cancer. A 3.5 cm umbilical incision was made, and SILS was performed using a single-port surgical device with three 5 mm trocars placed in the incision. The sigmoid colon was resected with a linear stapler, which required switching from a 5 mm trocar to a 12 mm trocar. Laparoscopic anastomosis was performed using the double-stapling technique. The second case involved an 81-year-old man with dual cancers located in the sigmoid colon and lower rectum, 8 cm from the anal verge. The abdominal approach was performed using SILS, similar to the first case, along with a transanal total mesorectal excision (TaTME) from the perineum by 2 teams. Anastomosis was performed laparoscopically using a single-stapling technique. Neither patient experienced postoperative complications, and both remained free of recurrence at 42 and 7 months, respectively.

**CONCLUSIONS:**

SILS is a feasible approach for patients with sigmoid colon cancer or rectal cancer and SIT.

## Abbreviations

AV
: anal verge
CT
: computed tomography
SILS
: single-incision laparoscopic surgery
SIT
: situs inversus totalis
TaTME
: transanal total mesorectal excision
TME
: total mesorectal excision

## INTRODUCTION

Situs inversus totalis (SIT) is a rare congenital condition characterized by the complete inversion of all thoracic and abdominal viscera. The incidence of SIT is estimated to be between 1 in 8000 and 25000.^1,2)^ Due to the mirror-image transposition of organs and associated vascular abnormalities, surgical procedures for patients with SIT are considered more challenging than for those without the condition, particularly in laparoscopic surgery.^3–5)^

Recently, single-incision laparoscopic surgery (SILS) has been described for patients with SIT.^6–8)^ While colorectal resections have been reported, there are no documented cases of procedures requiring intracavitary mesenteric dissection or anastomosis, such as those for sigmoid colon or rectal cancer. To the best of our knowledge, this is the first case report of sigmoid colon and rectal cancer in patients with SIT successfully treated with SILS.

## CASE PRESENTATION

### Case 1

**Fig. 1:**
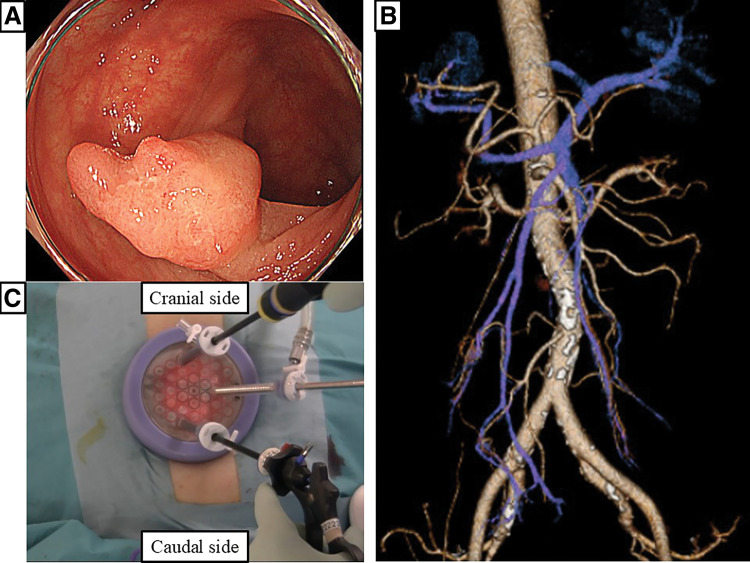
(**A**) Colonoscopic image: A superficial neoplastic lesion in the sigmoid colon. (**B**) Computed tomography angiography demonstrating complete transposition of the abdominal viscera. (**C**) Three 5 mm trocars inserted into the multiport access device through a 3.5 cm umbilical incision.

A 64-year-old woman was admitted with occult blood in her stool. Colonoscopy revealed a sigmoid colon tumor approximately 12 mm in size. Endoscopic mucosal resection was performed, confirming a well-differentiated adenocarcinoma with 600 µm of infiltration into the submucosa. Computed tomography (CT) revealed a complete transposition of the abdominal viscera, confirming SIT (**Figs. 1A** and **1B**). There was no history of abdominal surgery.

#### Surgical procedures

**Fig. 2:**
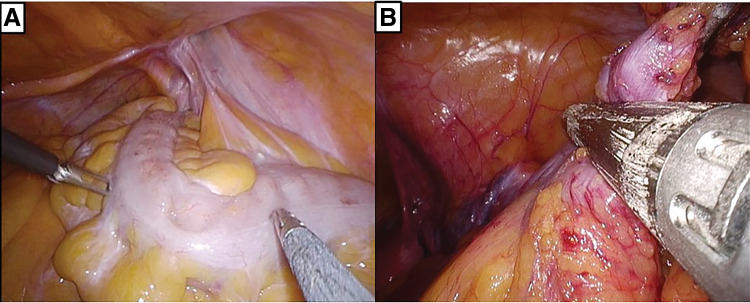
(**A**) The sigmoid colon was located on the patient’s right side. (**B**) The sigmoid colon resection performed with a linear stapler.

The patient was placed in the Trendelenburg position under general anesthesia. We used the Smart Retractor (TOP Corporation, Tokyo, Japan) and a multiport access device (Free Access; TOP Corporation) for the initial 3.5 cm umbilical incision. Three 5 mm trocars were inserted into the multiport access device (**Fig. 1C**). A 30°, 5 mm rigid laparoscope was used with CO_2_ pneumoperitoneum at 10 mmHg. The surgeon performed the operation standing on the left side of the patient. During laparoscopy, the sigmoid colon was found on the right side, and there were severe adhesions between the greater omentum and the sigmoid colon (**Fig. 2A**). The sigmoid colon was mobilized using a lateral approach. The mesorectum and sigmoid mesocolon were mobilized by connecting the medial and lateral sides. Complete mesocolic excision was performed with occasional nondominant manipulation. Nondominant manipulation was mainly required when performing dissection or central vessel ligation (CVL) toward the patient’s head side. CVL was achieved by resecting the vessels, including the inferior mesenteric artery and vein, as well as the left colic artery. After confirming tattooing on the serosa of the sigmoid colon, the mesentery was divided, and the sigmoid colon was resected with a linear stapler (changing the trocar from 5 to 12 mm) (**Fig. 2B**). In SILS, interference with the forceps or stapler was reduced when the scope was in the far view. The specimen was removed through the umbilical incision. Laparoscopic anastomosis was performed using the double-stapling technique. The operative time was 195 min. Pathological examination revealed no residual tumors or lymph node metastases. The patient was discharged on postoperative day 16 without complications. During a follow-up period of 42 months, she remained free of recurrence.

### Case 2

**Fig. 3:**
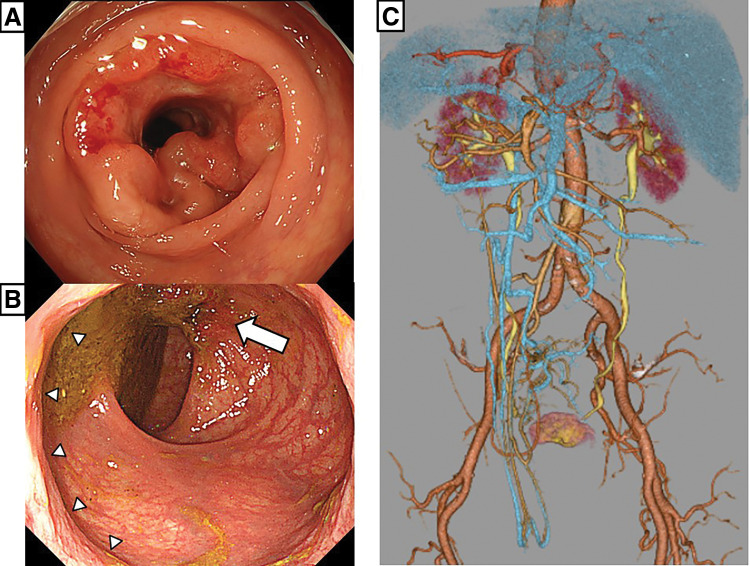
(**A**) Colonoscopic image: An ulcerofungating-type (Borrmann type II) tumor in the sigmoid colon. (**B**) An ulcerofungating-type (Borrmann type II) tumor in the lower rectum. (**C**) Computed tomography angiography showing complete transposition of the abdominal viscera. White arrow: lower rectal tumor; white triangles: upper edge of the anal canal.

An 81-year-old man was admitted to our hospital with melena. Colonoscopy revealed a complete circumferential tumor of the sigmoid colon and an ulcerofungating-type (Borrmann type II) tumor, measuring 20 mm in the lower rectum and located 8 cm from the anal verge (AV). Both tumors were diagnosed as well-differentiated adenocarcinomas upon biopsy. CT revealed a complete transposition of the abdominal viscera, confirming SIT (**Fig. 3**). There was no history of abdominal surgery.

#### Surgical procedures

**Fig. 4:**
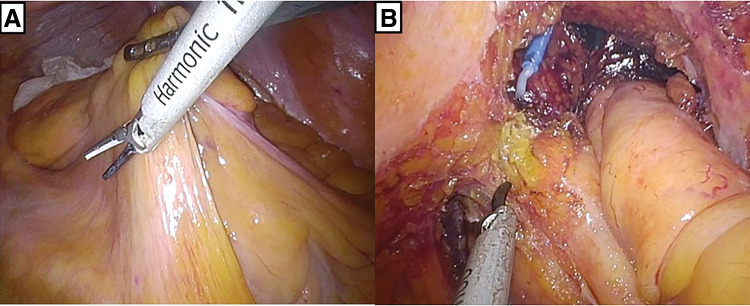
(**A**) When mobilizing the left side of the mesorectum, the incision was made with the hands crossed. (**B**) Dissection of the left side of the rectum, performed simultaneously from the abdomen and perineum.

We performed laparoscopic surgery using both abdominal and transanal approaches by 2 teams simultaneously (**Fig. 4**). The abdominal approach was executed using the same technique as in Case 1. In the pelvis, the rectum can be mobilized even with solo surgery by elevating the peritoneal reflection with sutures. The specimen was removed through an umbilical incision. Anastomosis was performed laparoscopically using a single-stapling technique. The operative time was 228 min. Pathological examination showed that the rectal lesion infiltrated the submucosa, while the sigmoid colon lesion infiltrated the subserosa. No lymph node metastasis was observed. The patient was discharged on postoperative day 15 without complications. During the follow-up period of 7 months, the patient remained free of recurrence.

## DISCUSSION

SIT is a rare congenital abnormality in which all thoracic and abdominal organs are transposed from their normal anatomical positions to the opposite side of the body. Surgical procedures in patients with SIT are technically more challenging due to the altered anatomical locations of the organs, particularly during laparoscopic surgery. Laparoscopic procedures in patients with SIT have been reported for colorectal resection, as well as for gastrectomy, pancreaticoduodenectomy, appendectomy, and cholecystectomy.^3,4,9–11)^ Laparoscopic surgery for colorectal cancer has also been documented; careful consideration of trocar positioning and adjustments to the surgeon’s position are necessary in patients with SIT, as most surgeons are right-handed (**Table 1**).^3,12–14)^

**Table 1:** Summary of cases of laparoscopic colorectal cancer surgery for situs inversus totalis in the English literature

Types of laparoscopic surgery	Author	Year	Tumor location	Operation time (min)	Blood loss (mL)	Complications	Location of the anastomosis
(SILS) Ileocolectomy	Hirano et al.^7)^	2015	Cecum	125	Minimal	None	Extracorporeal
Right hemicolectomy	Fujiwara et al.^21)^	2007	Ascending colon	191	60	None	Extracorporeal
	Kim et al.^14)^	2011	Ascending colon	119	Minimal	None	Extracorporeal
	Sasaki et al.^22)^	2017	Ascending colon	109	10	None	Extracorporeal
	Kojima et al.^23)^	2019	Ascending colon	237	20	None	Extracorporeal
	Hu et al.^24)^	2022	Ascending colon	178	50	None	Extracorporeal
Colectomy	Sakamoto et al.^25)^	2022	Transverse colon	257	Minimal	None	Intracorporeal
Left hemicolectomy	Sumi et al.^26)^	2013	Transverse colon	402	230	None	Extracorporeal
	Zheng et al.^27)^	2022	Descending colon	240	50	None	Extracorporeal
Sigmoidectomy	Yaegashi et al.^12)^	2015	Sigmoid colon	189	13	None	Intracorporeal
	Takeda et al.^3)^	2019	Sigmoid colon	195	Minimal	None	Intracorporeal
	Chen et al.^28)^	2020	Sigmoid colon	195	Minimal	None	Intracorporeal
	Kudo et al.^29)^	2022	Sigmoid colon	243	Minimal	None	Intracorporeal
(SILS) Sigmoidectomy	Our Case 1	2025	Sigmoid colon	195	110	None	Intracorporeal
Total mesorectal excision	Huh et al.^30)^	2010	Lower rectum	250	120	None	Intracorporeal
(SILS) Total mesorectal excision	Our Case 2	2025	Sigmoid colon Lower rectum	228	Minimal	None	Intracorporeal
Abdominoperineal resection	Choi et al.^13)^	2011	Lower rectum	325	300	None	–

SILS, single-incision laparoscopic surgery

SILS is technically challenging; however, it offers potential benefits such as improved cosmesis and reduced postoperative pain due to fewer scars. SILS for colorectal cancer is believed to yield good short- and long-term outcomes.^15–17)^ We previously reported that SILS colorectal resection can be successfully performed using a modified approach, even by surgeons who are not experts in laparoscopic surgery.^17)^ Additionally, SILS for SIT has been successfully performed for colorectal resection.^7)^ We believe that SILS is particularly suitable for patients with SIT, provided that trocar positioning is carefully considered. The multiport access device can be rotated, allowing for adjustments in the angle of the forceps for each specific task. The use of staplers with variable angles and the occasional crossing of the forceps and stapler facilitates the resection of the sigmoid colon and upper rectum, as demonstrated in Case 1.

The rate of incomplete total mesorectal excision (TME) is approximately 10%.^18)^ Since these data include non-laparoscopic procedures, it is possible that laparoscopic techniques can be performed with greater precision. In SILS, mobilization of the mesorectum can be achieved by altering the manipulation angle; however, the forceps move tangentially to the mesorectum, which complicates proper intraperitoneal resection of the mesorectum. This could increase the likelihood of incomplete TME, making it difficult to achieve an adequate distal resection margin.^19)^ Transanal TME (TaTME) helps obtain high-quality specimens and lowers the rates of positive distal and circumferential resection margins, which can significantly impact patient prognosis.^20)^ As the rectum is not affected by left-right organ inversion in patients with SIT, we believe that the difficulty of performing adequate mesorectal resection in SILS is not particularly high in these patients. SILS for rectal cancer is usually performed by the surgeon standing on the patient’s right side, whereas in patients with SIT, the surgeon stands on the patient’s left side. As most surgeons are right-handed, mobilization of the mesorectum in SILS is even considered rather suitable in patients with SIT, as the manipulation is to the left side for the surgeon. However, SILS resection is challenging in cases of lower rectal cancer, where the tumor’s lower edge is located below the peritoneal reflection, as seen in Case 2. With TaTME, perineal manipulation can be performed closer to the peritoneal reflection, making mobilization of the lower rectum and mesorectal dissection unnecessary. Consequently, TME for lower rectal cancer in SILS for SIT was successfully achieved using TaTME.

## CONCLUSION

We report the first case of SILS in a patient with SIT and sigmoid colon and/or rectal cancer. While SILS is technically challenging, with careful planning and ingenuity, colorectal resection can be successfully performed in patients with SIT. Furthermore, SILS may be particularly well-suited for colorectal mobilization in these cases.

## ACKNOWLEDGMENTS

None.

## DECLARATIONS

### Funding

This report did not receive any specific grants from funding agencies in the public, commercial, or not-for-profit sectors.

### Authors’ contributions

Conception and study design: MM.

Acquisition of data: MM, KT, SK, YO, SS, DS, SHa, YS, MK, YK, KK, and SO.

Analysis and/or interpretation of data: MM.

Drafting the manuscript: MM.

Revising the manuscript critically for important intellectual content: MM and SHi.

Approval of the final version of the manuscript to be published: MM, KT, SK, YO, SS, DS, SHa, YS, MK, YK, KK, SO, and SHi.

Consent to be responsible for this research: MM, KT, SK, YO, SS, DS, SHa, YS, MK, YK, KK, SO, and SHi.

### Availability of data and materials

The datasets supporting the conclusions of this article are included within the article and its additional files.

### Ethics approval and consent to participate

Ethics Committee approval was not required for this manuscript. The participants provided informed consent, and their anonymity was preserved.

### Consent for publication

These patients consented to the reporting of these cases in a scientific publication.

### Competing interests

The authors declare that they have no competing interests.

## References

[1] Peeters H Devriendt K. Human laterality disorders. Eur J Med Genet 2006; 49: 349–62. 16461029 10.1016/j.ejmg.2005.12.003

[2] Coronel M Lanke G Cambell D Performing endoscopic retrograde cholagiopancreatography and endoscopic ultrasound for management of malignant bile duct obstruction in a patient with a situs inversus totalis. ACG Case Rep J 2020; 7: e00483. 33324708 10.14309/crj.0000000000000483 PMC7725251

[3] Takeda T Haraguchi N Yamaguchi A Laparoscopic sigmoidectomy in a case of sigmoid colon cancer with situs inversus totalis. Asian J Endosc Surg 2019; 12: 111–3. 29601667 10.1111/ases.12483 PMC6585653

[4] Golash V. Laparoscopic management of acute appendicitis in situs inversus. J Minim Access Surg 2006; 2: 220–1. 21234150 10.4103/0972-9941.28184 PMC3016484

[5] Palanivelu C Rangarajan M John SJ Laparoscopic appendectomy for appendicitis in uncommon situations: the advantages of a tailored approach. Singapore Med J 2007; 48: 737–40. 17657381

[6] Rajkumar JS Syed A Anirudh JR Single-incision multi-port appendectomy for a patient with situs inversus totalis: first case report. Sultan Qaboos Univ Med J 2016; 16: e242–5. 27226919 10.18295/squmj.2016.16.02.018 PMC4868527

[7] Hirano Y Hattori M Douden K Single-incision laparoscopic surgery for colon cancer in patient with situs inversus totalis: report of a case. Indian J Surg 2015; 77: 26–8. 25972634 10.1007/s12262-014-1075-9 PMC4425779

[8] Lee IY Lee D Lee CM. Case report: single-port laparoscopic total gastrectomy for gastric cancer in patient with situs inversus totalis. Front Oncol 2023; 13: 1094053. 36741026 10.3389/fonc.2023.1094053 PMC9889819

[9] Kigasawa Y Takeuchi H Kawakubo H Laparoscopy-assisted distal gastrectomy in a case of gastric cancer with situs inversus totalis: a case report. Asian J Endosc Surg 2017; 10: 47–50. 27739194 10.1111/ases.12326

[10] Hussan MA Yang Z Dong X A laparoscopic pancreaticoduodenectomy for pancreatic adenocarcinoma in a patient with situs inversus totalis. J Surg Case Rep 2021; 2021: rjab316. 34316352 10.1093/jscr/rjab316 PMC8301639

[11] Oms LM Badia JM. Laparoscopic cholecystectomy in situs inversus totalis: the importance of being left-handed. Surg Endosc 2003; 17: 1859–61. 14959744 10.1007/s00464-003-9051-7

[12] Yaegashi M Kimura T Sakamoto T Laparoscopic sigmoidectomy for a patient with situs inversus totalis: effect of changing operator position. Int Surg 2015; 100: 638–42. 25875545 10.9738/INTSURG-D-14-00217.1 PMC4400931

[13] Choi SI Park SJ Kang BM Laparoscopic abdominoperineal resection for rectal cancer in a patient with situs inversus totalis. Surg Laparosc Endosc Percutan Tech 2011; 21: e87–90. 21471789 10.1097/SLE.0b013e31820b0258

[14] Kim HJ Choi GS Park JS Laparoscopic right hemicolectomy with D3 lymph node dissection for a patient with situs inversus totalis: report of a case. Surg Today 2011; 41: 1538–42. 21969158 10.1007/s00595-010-4530-7

[15] Katsuno G Fukunaga M Nagakari K Short-term and long-term outcomes of single-incision versus multi-incision laparoscopic resection for colorectal cancer: a propensity-score-matched analysis of 214 cases. Surg Endosc 2016; 30: 1317–25. 26139507 10.1007/s00464-015-4371-y

[16] Watanabe J Ishibe A Suwa H Long-term outcomes of a randomized controlled trial of single-incision versus multi-port laparoscopic colectomy for colon cancer. Ann Surg 2021; 273: 1060–5. 33630448 10.1097/SLA.0000000000004252

[17] Miyasaka M Kitashiro S Takahashi M Long-term outcomes of single-incision laparoscopic colectomy for right-sided colon cancer utilising a craniocaudal approach. J Minim Access Surg 2024; 20: 408–13. 38214348 10.4103/jmas.jmas_191_23 PMC11601954

[18] Matsubara N Miyata H Gotoh M Mortality after common rectal surgery in Japan: a study on low anterior resection from a newly established nationwide large-scale clinical database. Dis Colon Rectum 2014; 57: 1075–81. 25101603 10.1097/DCR.0000000000000176

[19] Kwak JY Yang KM Han MS. Feasibility of single-incision plus one port laparoscopic low anterior resection for rectal cancer. J Minim Invasive Surg 2020; 23: 120–5. 35602382 10.7602/jmis.2020.23.3.120 PMC8985631

[20] Roodbeen SX Spinelli A Bemelman WA Local recurrence after transanal total mesorectal excision for rectal cancer: a multicenter cohort study. Ann Surg 2021; 274: 359–66. 31972648 10.1097/SLA.0000000000003757

[21] Fujiwara Y Fukunaga Y Higashino M Laparoscopic hemicolectomy in a patient with situs inversus totalis. World J Gastroenterol 2007; 13: 5035–7. 17854150 10.3748/wjg.v13.i37.5035 PMC4434631

[22] Sasaki K Nozawa H Kawai K Laparoscopic hemicolectomy for a patient with situs inversus totalis: a case report. Int J Surg Case Rep 2017; 41: 93–6. 29055878 10.1016/j.ijscr.2017.10.011 PMC5651547

[23] Kojima Y Sakamoto K Tomiki Y Laparoscopic right colectomy for a patient with situs inversus totalis. J Surg Case Rep 2019; 2019: rjz080. 30949333 10.1093/jscr/rjz080 PMC6439510

[24] Hu JL Li QY Wu K. Ascending colon cancer and situs inversus totalis-altered surgeon position for successful laparoscopic hemicolectomy: a case report. World J Clin Oncol 2022; 13: 848–52. 36337311 10.5306/wjco.v13.i10.848 PMC9630992

[25] Sakamoto J Ozawa H Nakanishi H Laparoscopic colectomy for a patient with situs inversus totalis: the usefulness of preoperative assessment. Am J Case Rep 2022; 23: e935538. 35869611 10.12659/AJCR.935538 PMC9326785

[26] Sumi Y Tomono A Suzuki S Laparoscopic hemicolectomy in a patient with situs inversus totalis after open distal gastrectomy. World J Gastrointest Surg 2013; 5: 22–6. 23515492 10.4240/wjgs.v5.i2.22 PMC3600568

[27] Zheng ZL Zhang SR Sun H Laparoscopic radical resection for situs inversus totalis with colonic splenic flexure carcinoma: a case report. World J Clin Cases 2022; 10: 5435–40. 35812688 10.12998/wjcc.v10.i16.5435 PMC9210886

[28] Chen W Liang JL Ye JW Laparoscopy-assisted resection of colorectal cancer with situs inversus totalis: a case report and literature review. World J Gastrointest Endosc 2020; 12: 310–6. 32994862 10.4253/wjge.v12.i9.310 PMC7503618

[29] Kudo T Matsuda T Urakawa N Laparoscopic sigmoidectomy with splenic flexure mobilization for colon cancer in situs inversus totalis: preoperative assessment and preparation. Asian J Endosc Surg 2022; 15: 168–71. 33893717 10.1111/ases.12944

[30] Huh JW Kim HR Cho SH Laparoscopic total mesorectal excision in a rectal cancer patient with situs inversus totalis. J Korean Med Sci 2010; 25: 790–3. 20436720 10.3346/jkms.2010.25.5.790 PMC2858843

